# Novel Anti-B-cell Maturation Antigen Alpha-Amanitin Antibody-drug Conjugate HDP-101 Shows Superior Activity to Belantamab Mafodotin and Enhanced Efficacy in Deletion 17p Myeloma Models

**DOI:** 10.21203/rs.3.rs-3843028/v1

**Published:** 2024-01-11

**Authors:** Ram Kumar Singh, Richard J. Jones, Fazal Shirazi, Li Qin, Jianxuan Zou, Samuel Hong, Hua Wang, Hans C. Lee, Krina K. Patel, Jie Wan, Rajan Kumar Choudhary, Isere Kuiatse, Andreas Pahl, Robert Z. Orlowski

**Affiliations:** The University of Texas MD Anderson Cancer Center; The University of Texas MD Anderson Cancer Center; The University of Texas MD Anderson Cancer Center; The University of Texas MD Anderson Cancer Center; The University of Texas MD Anderson Cancer Center; The University of Texas MD Anderson Cancer Center; The University of Texas MD Anderson Cancer Center; The University of Texas MD Anderson Cancer Center; The University of Texas MD Anderson Cancer Center; The University of Texas MD Anderson Cancer Center; The University of Texas MD Anderson Cancer Center; The University of Texas MD Anderson Cancer Center; Heidelberg Pharma AG; The University of Texas MD Anderson Cancer Center

**Keywords:** HDP-101, p53, Deletion 17p, proteasome capacity, proteasome inhibitor sensitivity

## Abstract

B-cell maturation antigen (BCMA) plays a pathobiologic role in myeloma and is a validated target with five BCMA-specific therapeutics having been approved for relapsed/refractory disease. However, these drugs are not curative, and responses are inferior in patients with molecularly-defined high-risk disease, including those with deletion 17p (del17p) involving the tumor suppressor *TP53*, supporting the need for further drug development. Del17p has been associated with reduced copy number and gene expression of RNA polymerase II subunit alpha (*POLR2A*) in other tumor types. We therefore studied the possibility that HDP-101, an anti-BCMA antibody drug conjugate (ADC) with the POLR2A poison α-amanitin could be an attractive agent in myeloma, especially with del17p. HDP-101 reduced viability in myeloma cell lines representing different molecular disease subtypes, and overcame adhesion-mediated and both conventional and novel drug resistance. After confirming that del17p is associated with reduced *POLR2A* levels in publicly available myeloma patient databases, we engineered *TP53* wild-type cells with a *TP53* knockout (KO), *POLR2A* knockdown (KD), or both, the latter to mimic del17p. HDP-101 showed potent anti-myeloma activity against all tested cell lines, and exerted enhanced efficacy against POLR2A KD and dual *TP53* KO/POLR2A KD cells. Mechanistic studies showed HDP-101 up-regulated the unfolded protein response, activated apoptosis, and induced immunogenic cell death. Notably, HDP-101 impacted CD138-positive but not-negative primary cells, showed potent efficacy against aldehyde dehydrogenase-positive clonogenic cells, and eradicated myeloma in an *in vivo* cell line-derived xenograft (CDX). Interestingly, in the CDX model, prior treatment with HDP-101 precluded subsequent engraftment on tumor cell line rechallenge in a manner that appeared to be dependent in part on natural killer cells and macrophages. Finally, HDP-101 was superior to the BCMA-targeted ADC belantamab mafodotin against cell lines and primary myeloma cells *in vitro*, and in an *in vivo* CDX. Together, the data support the rationale for translation of HDP-101 to the clinic, where it is now undergoing Phase I trials, and suggest that it could emerge as a more potent ADC for myeloma with especially interesting activity against the high-risk del17p myeloma subtype.

## Introduction

Multiple myeloma is a clonal plasma cell malignancy characterized clinically in symptomatic patients by hypercalcemia, renal insufficiency, anemia, painful bony lesions, and an increased risk of infectious complications. Several new drug classes have been introduced over the last two decades, including proteasome inhibitors (PIs), immunomodulatory drugs (IMiDs), and monoclonal antibodies (mAbs) to cell surface targets such as CD38 and Signaling lymphocytic activation molecule family member 7 (1). These have added to the effectiveness of older therapeutics such as corticosteroids and alkylating agents, and use of these drugs in rational combination regimens has substantially improved long-term outcomes. Encouragingly, this trend seems likely to continue with the development of novel immunotherapeutics that work through various mechanisms of action, including antibody drug conjugates (ADCs)(2), T-cell engaging bispecific antibodies (3), and chimeric antigen receptor (CAR)-guided T-cells (4). B-cell maturation antigen (BCMA) has been an especially popular target for these drugs since it is commonly and fairly specifically expressed on plasma cells, and plays an important role in plasma cell differentiation (5). Moreover, binding of the BCMA ligands A proliferation-inducing ligand (APRIL) and B-cell-activating factor (BAFF) promotes myeloma cell proliferation and survival, as well as immune suppression in the tumor microenvironment (5), further contributing to disease pathobiology. However, not all patients benefit equally from BCMA-targeted agents, and this is especially true for those with molecularly or clinically defined high-risk disease (6, 7). For example, lower response rates were noted among high-risk patients in the pivotal trials of the ADC belantamab mafodotin (8), the bispecific teclistamab (9), and the CAR T-cell product idecabtagene vicleucel (10), which were the first drugs in each mechanistic category to achieve regulatory approvals. Thus, novel strategies and drugs to overcome the adverse prognosis of patients with high-risk features are still needed.

The high-risk disease category encompasses several unique molecular subtypes of myeloma (6, 7), and patients with deletion of 17p (del17p) consistently represent one of these that have inferior long-term outcomes (11, 12, 13, 14, 15, 16). While the proportion of myeloma patients with del17p is approximately 10% when the disease is newly diagnosed, it then increases substantially in the relapsed/refractory setting, especially with more advanced and aggressive variants such as plasma cell leukemia (17, 18, 19, 20). *TP53* loss is felt to be the pathognomonic lesion in del17p myeloma patients, but neighboring genes may also be co-deleted. One example is RNA polymerase II subunit A (*POLR2A*), which is located about 200 kilobases from *TP53*, and has been described to be co-deleted in a large proportion of colorectal cancer cases (21). Decreased *POLR2A* expression through such haploinsufficiency may enhance sensitivity to α-amanitin (21, 22), a cyclic peptide that targets POLR2A at low concentrations (23). This provided us with the rationale to further evaluate HDP-101 (24, 25, 26), a novel ADC with a structural derivative of α-amanitin optimized for stability conjugated to an engineered cysteine at position 265, and linked to a BCMA-targeted antibody through a Cathepsin B-cleavable linker.

In the current line of research, we developed pre-clinical models of *TP53*-wild type and deleted myeloma, both with and without *POLR2A* suppression, and found that HDP-101 showed potent anti-proliferative and pro-apoptotic activity especially in the former. HDP-101 overcame adhesion-mediated and novel drug resistance, and was equally effective against clonotypic cells defined by expression of Aldehyde dehydrogenase (*ALDH*). Mechanistic studies showed HDP-101 up-regulated the unfolded protein response (UPR) gene program, while proteomic studies confirmed induction of multiple cellular UPR arms, activation of apoptosis, and induction of immunogenic cell death (ICD). In cell line-derived xenograft models (CDXs) in immune-deficient mice, a single HDP-101 dose was sufficient to cure myeloma, and also to prevent tumor re-engraftment on subsequent tumor cell rechallenge, with evidence of a dependence on natural killer (NK) cells and macrophages. Finally, HDP-101 was superior to the previously approved BCMA-targeted ADC belantamab mafodotin against cell lines *in vitro* and in an *in vivo* del17p model. Together, the data further validate the efficacy of this new BCMA ADC, which is undergoing Phase I trials, and suggest it could be equipotent, or perhaps even more effective against myeloma with the high-risk del17p abnormality.

## Methods

### Key reagents

The anti-BCMA ADC HDP-101 (24, 25, 26) and control reagents were provided by Heidelberg Pharma AG (Ladenburg, Germany), while anti-Asialo-GM1 antibody was obtained from BioLegend (San Diego, CA). Bortezomib and the gamma secretase inhibitor (GSI) RO4929097 (27) were purchased from Selleck Chemical (Houston, TX), and drug stock solutions were prepared in dimethyl-sulfoxide (Fisher Scientific; Pittsburgh, PA) and stored at −20°C.

### Primary patient samples and myeloma cell lines

Primary cells were collected under a protocol approved by the MD Anderson Institutional Review Board after informed consent was obtained in compliance with the Declaration of Helsinki. Human myeloma and the HS-5 (RRID:CVCL_3720) human stromal cell lines were maintained in RPMI 1640 medium (Corning Cellgro; Manassas, VA) supplemented with L-glutamine, 5% fetal bovine serum and 1% penicillin/streptomycin, as well as interleukin (IL)-6 in the case of ANBL-6 (RRID:CVCL_5425) and KAS-6/1 (RRID:CVCL_9544) cells. Drug-resistant myeloma lines were developed as described previously (28). Please refer to the Supplementary Methods section for additional details on this and other aspects of the techniques employed.

To develop a model of del17p myeloma, we started with *TP53* wild-type (WT) H929 (RRID:CVCL_1600), MM1.S (RRID:CVCL_8792), and MOLP-8 (RRID:CVCL_2124) cells. These were engineered to knockout (KO) *TP53* using either sequence-specific zinc-finger nucleases targeting exon 7 of the *TP53* DNA binding domain (MM1.S and MOLP-8), or by Clustered regularly interspaced short palindromic repeats (CRISPR)/CRISPR-associated protein 9 (Cas 9)-based (H929) genome editing (29). Both WT and KO cells were then subjected to stable *POLR2A* knockdown using shRNAs in the GIPZ vector from the MD Anderson Functional Genomics Core.

### Co-culture, viability, and apoptosis assays

Please consult the Supplementary Methods section for details on these assays, which were typically analyzed by flow cytometry (30).

### Western blotting, gene expression profiling, and real time polymerase chain reaction

Cell lysates were prepared, processed, and analyzed as detailed in the Supplementary Methods section, and Table S1 provides a list of the antibodies used. Please also consult this section for details on gene expression profiling analyses and real time PCR (qPCR).

### Xenograft modeling

These studies were performed following protocols approved through the Institutional Animal Care and Use Committee. Six- to 8-week-old female non-obese diabetic, severe combined immunodeficiency (NOD-SCID) mice, or NOD.Cg-Prkdc^scid^ IL2rγ^tm1Wjl^/SzJ (NSG) mice (The Jackson Laboratory; Bar Harbor, ME), were injected via tail vein with 1×10^6^ luciferase-labeled myeloma cells. Once weekly bioluminescent imaging was performed after administration of 10 μl/g D-luciferin substrate (Thermo Fisher Scientific) using the IVIS Spectrum In Vivo Imaging System (PerkinElmer; Waltham, MA). Treatment was initiated as detailed in the text, weekly imaging was performed and, if needed, mice were euthanized by CO_2_ inhalation per institutional guidelines.

### Statistical analyses

Data are represented as the mean plus either standard deviation (SD; for triplicate data from the same experiment) or standard errors of the mean (SEM; for multiple independent experiments). The significance of drug-effect relationships was determined by one-tailed unpaired t-tests or ANOVA using Graph-Pad Prism, and p < 0.05 was considered statistically significant.

## Results

### HDP-101 potently reduces viability of myeloma cells

**Figure 1:**
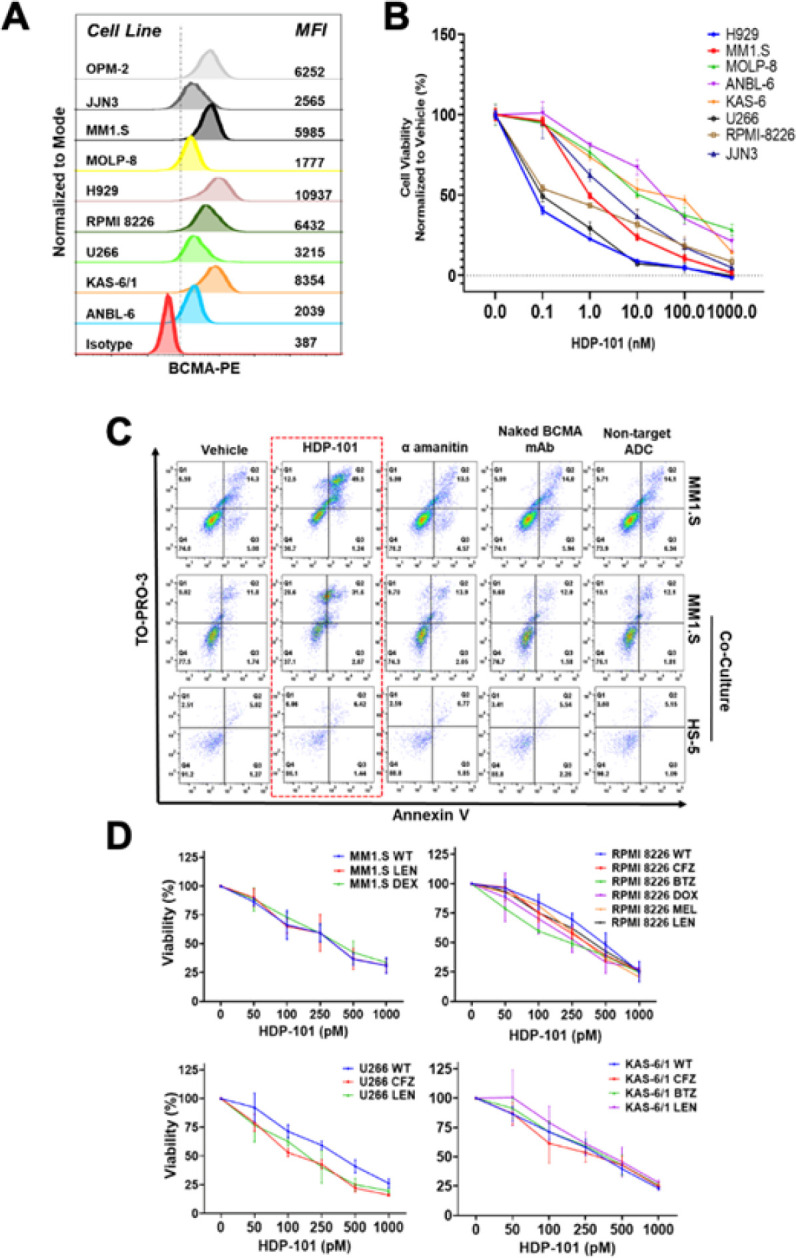
HDP-101 shows potent anti-myeloma activity in drug-naïve and drug-resistant cells. A panel of human-derived myeloma cell lines was assembled representing the t(4;14)(H929, KAS-6/1), t(11;14)(MOLP-8, U266), and t(14;16)(ANBL-6, JJN3 (RRID:CVCL_2078), MM1.S) subtypes. These also included *TP53*-WT (H929, MM1.S, MOLP-8) as well as IL-6-dependent cells (ANBL-6, KAS-6/1), and cells with 1q21 gain (3 copies; U266) and amplification (4 or more copies; ANBL-6, H929, JJN3, OPM-2 (RRID:CVCL_1625), and RPMI 8226). Cells were evaluated for their surface expression of BCMA by flow cytometry (**A**) compared to an isotype control, with results displayed as the mean fluorescence intensity (MFI). They were then exposed to the indicated HDP-101 concentrations for 72 hours (**B**), and viability was determined with the WST-1 tetrazolium assay. Data were plotted as viability compared with vehicle-treated cells arbitrarily set at 100%, and each point represents the mean of two independent experiments, each performed in triplicate. MM1.S myeloma cells were propagated either alone (**C**), or in a co-culture with HS-5 cells, and exposed to vehicle, HDP-101 (200 pM), the anti-BCMA HDP-101 antibody without conjugated a-amanitin (200 pM), or an anti-digoxigenin/a-amanitin ADC (200 pM), all for 48 hours. They were then analyzed for viability and apoptosis after TO-PRO-3 and Annexin V staining as detailed earlier. Drug-naïve and drug-resistant myeloma cell line models were exposed to the indicated HDP-101 concentrations for 96 hours (**D**), and viability was determined and is presented as detailed earlier. Note that wild-type MM.1S and RPMI 8226 cells are considered to be daratumumab-resistant.

To evaluate the activity of HDP-101, we studied a panel of BCMA-expressing myeloma cell lines (Fig. 1A) representing common molecular subtypes, including translocation (t)(11;14), t(4;14), t(14;16), gain and amplification of 1q, and different *TP53* status and IL-6 dependency. HDP-101 potently reduced viability in all of these lines in a dose-dependent manner (Fig. 1B), as evidenced by median inhibitory concentrations (IC_50_) ranging from 40 pM to 38.6 nM (Table S2). As the IC_50_ values across the cell lines did not correlate with surface BCMA expression (R^2^ = 0.094), we examined the impact of the GSI RO4929097 (27), which increased surface BCMA levels (Fig. S1A). When the GSI was added to HDP-101, the combination enhanced the reduction in viability compared with either agent alone (Fig. S1B). One prominent feature of myeloma clinically is the benefit that neoplastic cells receive from their interactions with the marrow microenvironment, including adhesion-mediated drug resistance (31). To model this, we co-cultured myeloma cells with Green fluorescent protein (GFP)-labeled HS-5 stromal cells, exposed these co-cultures to HDP-101 and control compounds, and evaluated them for Annexin V and TO-PRO-3 staining by gating on GFP-positive and-negative cells. HS-5 cells were essentially unaffected by HDP-101 as the viable (Annexin V^−^/TO-PRO-3^−^) cell fraction was stable under all tested conditions (Fig. 1C). In contrast, HDP-101 reduced the proportion of viable MM1.S myeloma cells to 36.7% from the baseline of 74.0%, while this fraction was 37.1% in the presence of HS-5 cells (Fig. 1C). Notably, free α-amanitin, the unconjugated BCMA antibody, and an anti-digoxigenin/α-amanitin control ADC did not impact viability. Qualitatively similar findings were noted when this same experiment was performed with H929 cells (Fig. S2). One especially relevant product of the microenvironment is APRIL, since it binds BCMA and contributes to multiple aspects of the transformed phenotype (5). Importantly, when excess APRIL or soluble BCMA were added, while there was slight interference with the activity of HDP-101 at low ADC concentrations (Fig. S3A-B), at higher, physiologically relevant levels, HDP-101 overcame these soluble factors. Finally, acquired drug resistance is another prominent aspect of myeloma, and agents that overcome resistance hold promise for relapsed/refractory disease. There was no difference between the efficacy of HDP-101 in MM1.S versus MM1.R (RRID:CVCL_8794) dexamethasone-resistant cells (Fig. 1D, left upper panel), which showed greater potency in these studies compared to Fig. 1B since cells were incubated for 96 hours, demonstrating also a time-dependent effect. Similarly, the sensitivity of RPMI 8226 cells (RRID:CVCL_0014) resistant to melphalan (RRID:CVCL_J434) or doxorubicin (RRID:CVCL_J431) was similar to (Fig. 1D), or even slightly higher than their WT counterparts (Table S3). Moving to models of novel drug resistance, HDP-101 activity was not reduced in bortezomib-resistant RPMI 8226 or KAS-6/1, or in carfilzomib-resistant RPMI 8226, U266 (RRID:CVCL_0566), or KAS-6/1 cells (Fig. 1D, Table S3). Finally, resistance to the cell-autonomous effects of lenalidomide was studied, and no reduction in efficacy was seen (Fig. 1D, Table S3).

### Del17p reduces POLR2A expression, increasing HDP-101 sensitivity

**Figure 2:**
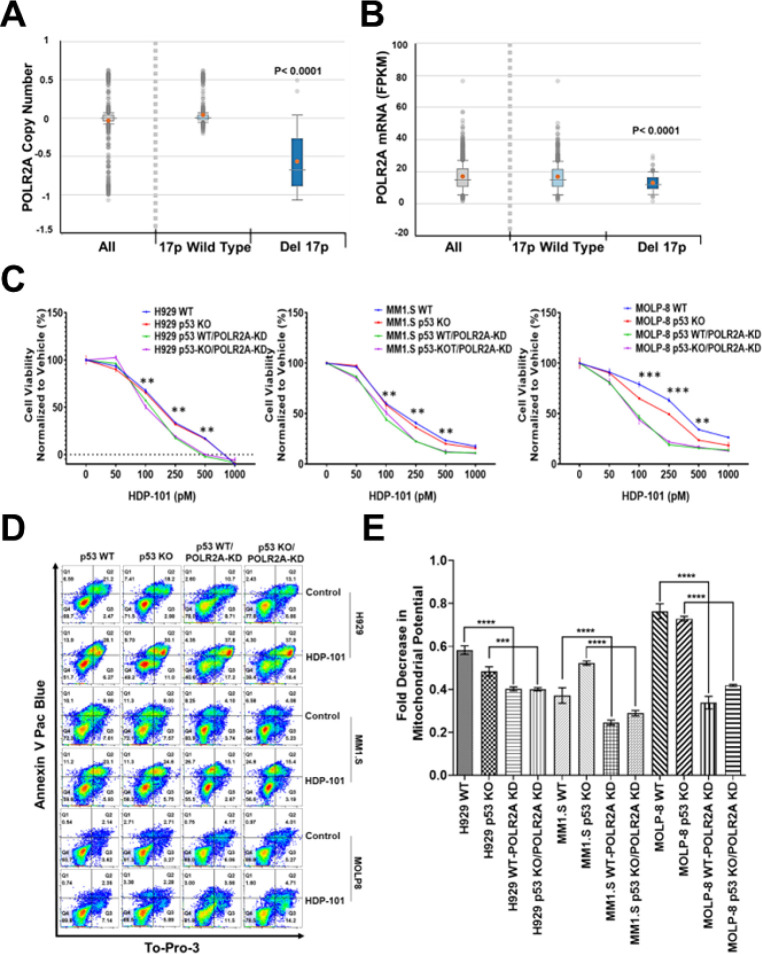
Efficacy of HDP-101 in *in vitro* del17p myeloma models. Analysis of the Multiple Myeloma Research Foundation CoMMpass^SM^ database showing the impact of del17p on *POLR2A* copy number (**A**) and mRNA expression (**B**) determined by seqFISH. Viability studies (**C**) were performed as detailed above in H929, MM1.S, and MOLP-8 cells that were either WT, *TP53* knock-out (KO), *POLR2A* knock-down (KD), or dual KO/KD. “**” indicate a p-value <0.01 while “***” indicates a p-value of <0.001 for the comparison of the *POLR2A* KD and dual KO/KD cells with their WT controls. To evaluate for apoptosis, MM1.S, H929 and MOLP-8 cells were examined by flow after staining for Annexin V and TO-PRO-3 (**D**), and for changes in their mitochondrial transmembrane potential (**E**).

Del17p producing *TP53* loss has been linked to *POLR2A* co-deletion and haploinsufficiency in colorectal and prostate carcinoma (21, 22). Therefore, we evaluated the Multiple Myeloma Research Foundation’s CoMMpass^SM^ database, and first confirmed that *POLR2A* copy number was reduced in del17p patients compared with those without del17p (mean 0.0373 vs. −0.5688, respectively, p < 0.0001; Fig. 2A). This was associated with a significant reduction in *POLR2A* mRNA levels by RNASeq (16.72978 FPKM (fragments per kilobase of exon model per million reads mapped) vs. 12.88536 FPKM, respectively, p < 0.0001; Fig. 2B). Notably, no change was seen in *BCMA* copy number and mRNA expression with del17p (Fig. S4). To model this *in vitro*, we started with *TP53* WT H929, MM1.S, and MOLP-8 cells and used genome editing to knockout (KO) *TP53* (Fig. S5A). Next, *POLR2A* was subjected to knockdown (KD) using shRNAs (Fig. S5B), and we exposed these to HDP-101. Compared to WT cells, *TP53* KO alone showed no impact on HDP-101 sensitivity in H929 and MM1.S cells, while there was a small degree of sensitization in MOLP-8 cells (Fig. 2C). In contrast, *POLR2A* KD alone had a more significant sensitizing effect and was comparable to that seen in the dual *TP53/POLR2A* KO/KD cells (Fig. 2C). Similarly, by Annexin V staining, HDP-101 induced evidence of decreased cell viability and increased apoptosis with increasing surface exposure of phosphatidyl-serine in the *POLR2A* KD and dual *TP53*/*POLR2A* KO/KD models (Fig. 2D). Finally, we also evaluated mitochondrial membrane potential changes, and the *TP53* WT/*POLR2A* KD and *TP53* KO/*POLR2A* KD cells both had substantially decreased potentials (Fig. 2E). In addition, cell cycle analysis was performed on the four H929-derived cell lines, and HDP-101 increased the apoptotic, sub-G0/G1 cell population to a greater extent in the two *POLR2A* KD models (Table S4). Together, the data support the statement that HDP-101 shows enhanced efficacy driven by reduced *POLR2A* levels, including in models of del17p myeloma, where sensitivity is increased compared to WT 17p controls.

### HDP-101 induces ER stress and ICD while reducing anti-apoptotic modulators

**Figure 3:**
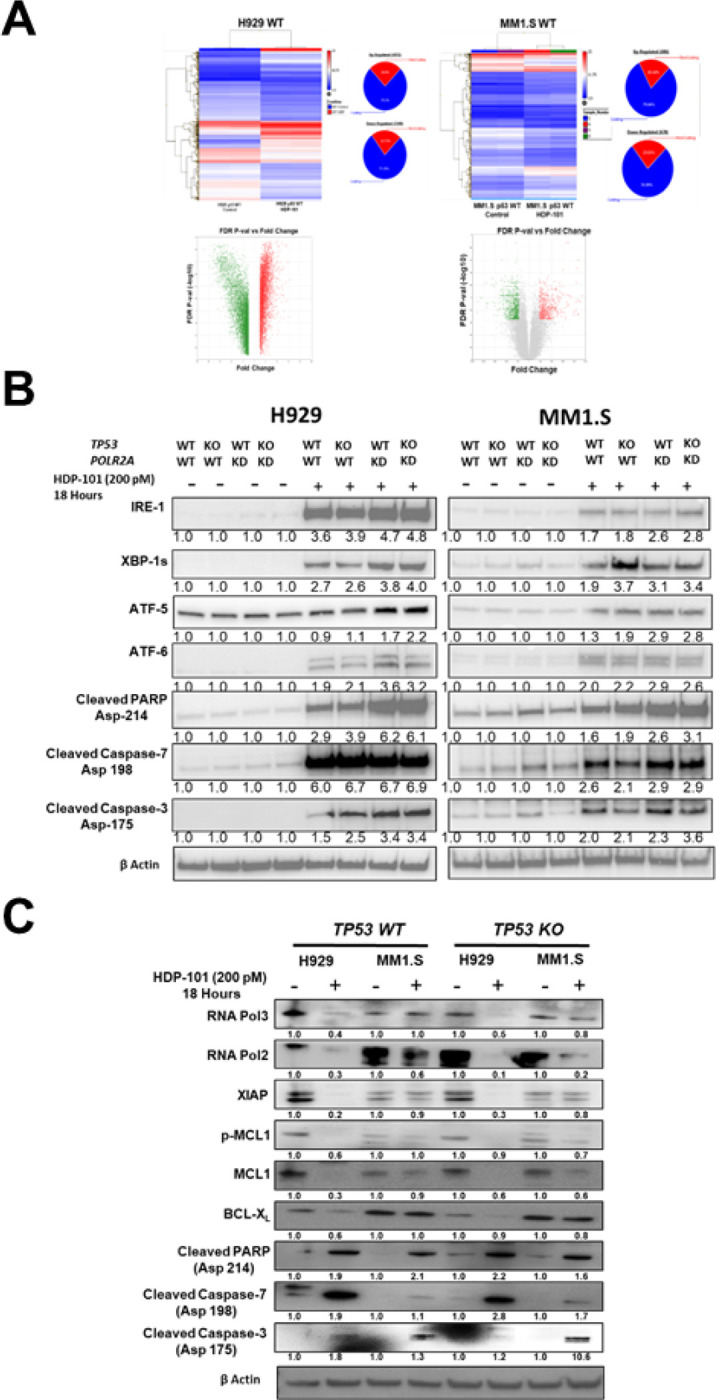
Molecular mechanism of action of HDP-101 via elevated ER stress. H929 and MM1.S *TP53* WT and KO cells were treated with HDP-101 at 200 pM for 24 hours, subjected to gene expression profiling, and the latter were analyzed to generate heat maps and volcano plots (**A**) of up- (red) or down- (blue or green) regulated genes. Extracts were then prepared and subjected to Western blotting to evaluate the abundance of proteins involved in the endoplasmic reticulum and mitochondrial unfolded protein response (**B**), as well as indicators of apoptosis such as cleaved caspases. Densitometry was performed to measure abundance with the controls set arbitrarily at 1.0, and the fold-increase with HDP-101 is shown from one of two independent experiments. These same extracts were then probed to look for changes in the abundance of key RNA polymerases and anti-apoptotic genes (C), as indicated in the text.

To gain mechanistic insights into the impact of HDP-101, gene expression analysis was performed on *TP53* WT H929 and MM1.S cells (Fig. 3A), and also on their *TP53* KO counterparts (Fig. S6). Pairwise analysis (Fig. S7) revealed that HDP-101 down-regulated more genes than were up-regulated, consistent with its mechanism as a *POLR2A* inhibitor. Enriched genes with a false discovery rate of < 5% are presented in Table 1 as hallmark gene sets (32) identified from the Molecular Signatures Database, with a focus on gene sets up-regulated in all four models. Notably, the UPR, a target of several therapeutics effective against myeloma (33), was induced by HDP-101. As this gene set contained Activating transcription factors (ATF)-5 and ATF6, as well as X-box protein (XBP)-1, we performed Western blotting to determine if there was evidence of UPR activation at the protein level. Both ATF6, which represents one arm of the ER UPR, and ATF5, which regulates the mitochondrial UPR, were induced by HDP-101 (Fig. 3B), and this tended to occurr to a greater extent in the *POLR2A* KD cell models. Similarly, Inositol-requiring enzyme 1 (IRE1) was induced, which led to enhanced downstream expression of the short isoform of XBP1 (Fig. 3B). On the down-regulated side, and consistent with the known mechanism of action of α-amanitin (34), RNA polymerase II and III levels were decreased (Fig. 3C). Notably, HDP-101 also decreased expression levels of several anti-apoptotic proteins, including Myeloma cell leukemia 1 (MCL1) and BCL-x_L_, as well as the X-linked inhibitor of apoptosis (Fig. 3C). These occurred in association with increased markers of apoptosis, including of cleaved poly-(ADP-ribose) polymerase (PARP1) and cleaved Caspases 3 and 7 (Fig. 3B,C).

**Figure 4:**
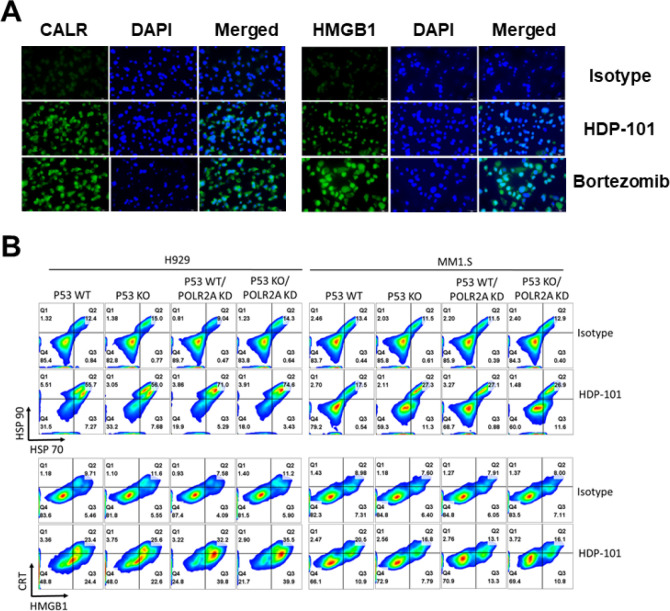
Immunogenic cell death as a mechanism of action of HDP-101. MM1.S cells were exposed to an isotype control antibody or to HDP-101, both at 100 pM for 48 hours, or to bortezomib at 1 nM as a appositive control. Immunofluorescence staining was then performed (**A**) to detect externalization of Calreticulin (CRT; left panel) or High mobility group box 1 (HMGB1; right panel). Cells were also stained with 4′,6-diamidino-2-phenylindole (DAPI) to detect regions of double stranded DNA. H929 and MM1.S models with WT *TP53, TP53* KO, *POLR2A*KD, or dual KO/KD were then treated as above and examined by flow cytometry (**B**) to look for cell surface CRT, HMGB1, and either Heat shock protein 70 (HSP70) or HSP90.

Several ADCs have the ability to induce both direct cytotoxic effects as well as ICD as part of their mechanisms of action (2) and, given the link between UPR activation and ICD (35), we next sought to determine if HDP-101 could so as well. Starting with the MM1.S cell model, isotype antibody or HDP-101-treated cells were analyzed to detect externalization of Calreticulin (CRT) or High mobility group box 1 (HMGB1) by immunofluorescence. Compared to the isotype negative control, HDP-101 enhanced cell surface levels of both CRT and HMGB1 (Fig. 4A), consistent with induction of ICD as seen with bortezomib as a positive control. Next, we expanded our panel to include H929 and MM1.S cells that were *TP53* WT, *TP53* KO, *POLR2A* KD, or dual KO/KD, and looked at CRT and HMGB1, as well as externalization of Heat shock protein 70 (HSP70) and HSP90. Once again compared to the isotype negative control, HDP-101 induced externalization of all four markers of ICD in both cell lines, with a trend seen towards increasing ICD in the H929 model system (Fig. 4B). Finally, we further broadened our analysis to look at our larger panel of myeloma cell lines representing multiple molecular subtypes of myeloma. Compared to the isotype control, HDP-101 enhanced cell surface levels of Calreticulin, HMGB1, and HSP70 in all of the cell lines (Fig. S8), suggesting consistent activation of the UPR and ICD.

### Activity of HDP-101 in vivo may engage NK cells and macrophages

**Figure 5:**
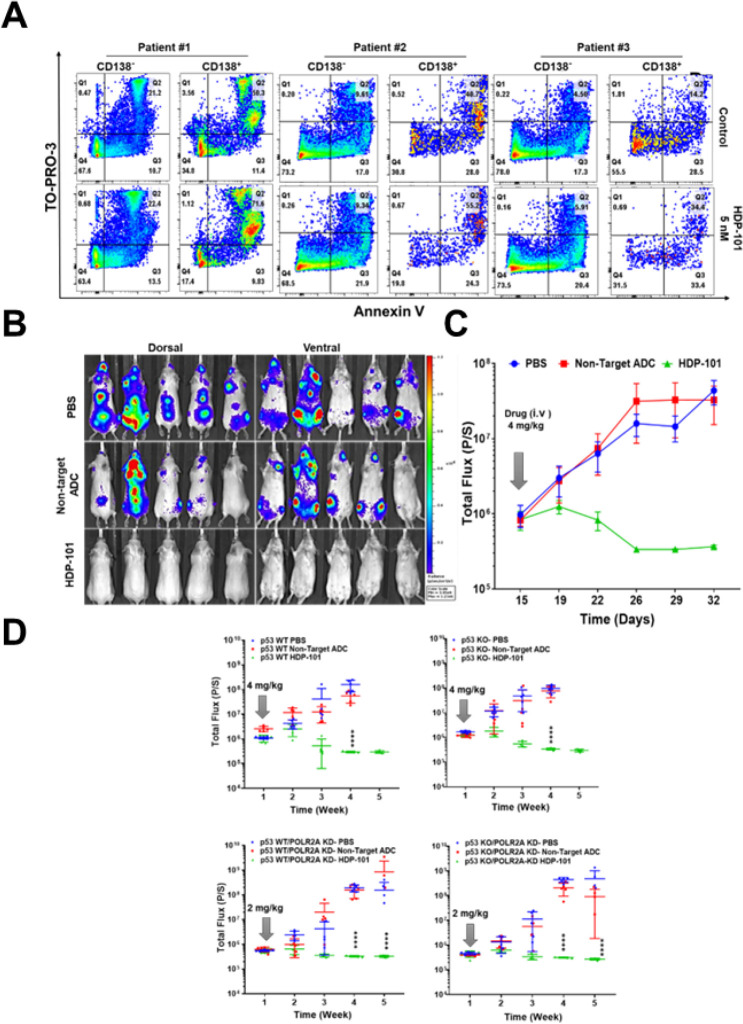
Efficacy of HDP-101 on primary samples and tumor xenograft. Three whole bone marrow aspirate samples from patients with relapsed/refractory myeloma were exposed to an isotype control or to HDP-101 at 5 nM for 24 hours. They were then analyzed by flow cytometry for their CD138 status with gating on myeloma (CD138^+^) and tumor microenvironment (CD138^−^) cells (**A**), and also for apoptosis by staining with Annexin V and TO-PRO-3. Luciferase (luc)-labeled MM1.S *TP53* WT cells were used to create xenografts in NOD/SCID mice and, after engraftment, they then received either the vehicle control, or either the anti-digoxigenin/a-amanitin ADC or HDP-101 at 4 mg/kg as a single dose. Serial *in vivo* whole animal imaging was performed, and is shown on day 30 with both ventral and dorsal views (**B**) and throughout the experiment indicating mean flux (**C**). Next, NOD-SCID mice were injected with 1×10^6^ luc-MM1.S *TP53* WT/*POLR2A* WT cells, *TP53* KO/*POLR2A* WT cells, *TP53* WT/*POLR2A* KD cells, or TP53 KO/POLR2A KD cells. When disease burden was measurable as a total flux between 5×10^5^ and 1×10^6^, mice were randomized (n=5 per cohort) based on flux to receive one dose of the PBS vehicle, HDP-101, or the control ADC at the indicated concentrations (D). They were then serially monitored for disease burden through week 5 by whole-animal *in vivo* imaging, with the mean flux graphed, along with the range of values. “****” indicates a p-value of <0.0001.

We next tested the ability of HDP-101 to exert effects against primary samples derived from patients, and separated these initially into fractions enriched for CD138-positive tumor cells and CD138-negative tumor microenvironment (TME) cells. HDP-101 showed minimal effects on the proportion of TME cells that were in the early apoptotic (Annexin V^+^/TO-PRO-3^−^), late apoptotic (Annexin V^+^/TO-PRO-3^+^), or necrotic (Annexin V^−^/TO-PRO-3^+^) stages of cell death (Fig. 5A) compared to the isotype control. In contrast, HDP-101 induced a substantial decrease in the viable cell fraction (Annexin V^−^/TO-PRO-3^−^) and an increase predominantly in the late apoptotic fraction under the condition tested (Fig. 5A). To examine the activity of HDP-101 *in vivo*, we developed a systemic xenograft using luciferase (luc)-labeled MM.1S WT *TP53* cells in NOD-SCID mice. Once engrafted, mice were randomized to produce three groups with equivalent disease burden to receive a single dose of vehicle, HDP-101 at 4 mg/kg, or the anti-digoxigenin/α-amanitin ADC control at 4 mg/kg. Compared to vehicle, the negative control/non-targeted α-amanitin-ADC did not substantially reduce disease burden (Fig. 5B,C). In contrast, HDP-101 first slowed disease progression, and this was followed by complete disease regression by day 32 (Fig. 5C). Similar patterns were seen in the MM1.S *TP53* KO, *POLR2A* KD, or dual KO/KD models, with regression of disease and no evidence of relapse even after a single 2 mg/kg dose of HDP-101 (Fig. 5D). Amanitins are able to impact quiescent cells (24, 25, 26) that could be equivalent to myeloma-initiating cells, which have been hypothesized as potentially contributing to drug resistance and disease relapse after other anti-myeloma therapies (36, 37). Therefore, we considered whether elimination of such cells could have contributed to the lack of relapse after HDP-101, and used staining for Aldehyde dehydrogenase (ALDH) to identify them (36). ALDH-positive cell fractions of H929 and MM.1S cells proved to be more clonogenic than their ALDH-negative counterparts (Fig. S9A,B). Notably, HDP-101 showed similar activity against the two, with no significant difference in the IC_50_ (Fig. S9C).

**Figure 6:**
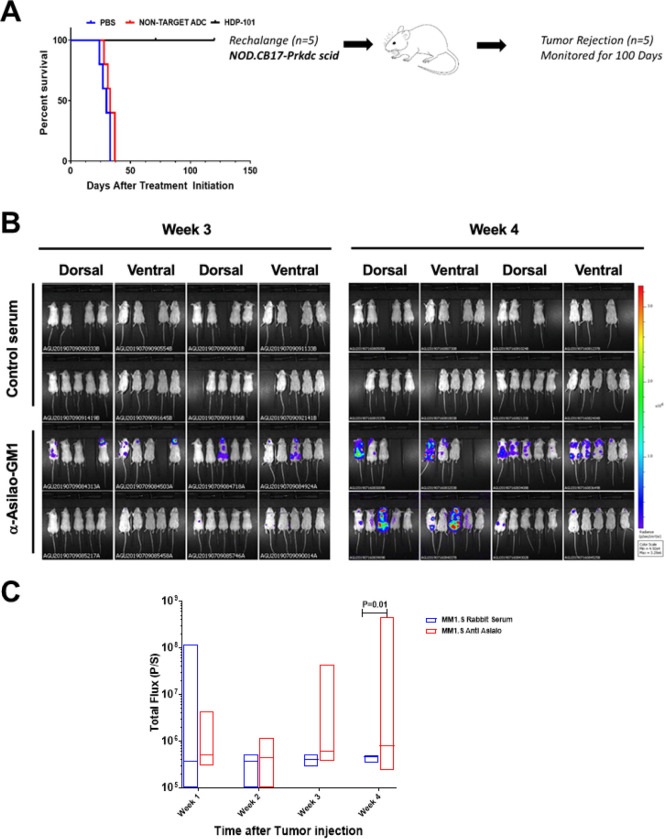
Prior HDP-101 treatment prevents subsequent tumor re-engraftment. Five NOD/SCID mice from the experiment in Figure 5B that showed no recurrence of tumor at day 100 of monitoring were rechallenged with a second batch of luc-MM1.S *TP53* WT cells (**A**) and monitored for disease engraftment for 50 days. As no engraftment was seen, this experiment was repeated and, at 100 days, mice were first given a dose of either control rabbit serum (n=17) or rabbit anti-Asilao-GM1 (n=19), followed by injection with luc-MM1.S *TP53* WT cells and monitored for disease burden. Whole animal imaging is shown for the entire cohorts at weeks 3 and 4 (**B**), and median fluorescent flux is shown for the entire cohorts in weeks 1 through 4 (**C**).

As disease relapse was not see in NOD-SCID mice xenografted with MM1.S cells and treated with HDP-101 at 100 days, we rechallenged a pilot cohort of five of these mice with luc-labeled MM1.S cells. Whereas engraftment was virtually 100% at baseline, none of these mice developed detectable tumor after another 100 day follow-up period (Fig. 6A). While these mice are immune deficient, recent re-evaluation of this model has indicated that they retain aspects of the innate immune system, including NK cells and macrophages (38). To determine if these could be contributing to the lack of re-engraftment, we repeated this experiment except that, just prior to disease re-challenge, we randomized mice to receive a dose of either control rabbit antiserum or a rabbit anti-Asialo-GM1 antibody. While the control group showed a range of fluorescence values after the initial MM1.S re-injection (Fig. 6B) consistent with some baseline circulating tumor cells, this signal rapidly disappeared (Fig. 6C). In contrast, treatment with anti-Asialo-GM1 to deplete NK cells and macrophages promoted tumor engraftment and led to a significantly higher disease burden (Fig. 6B,C).

### HDP-101 is more potent ADC than belantamab mafodotin

**Figure 7:**
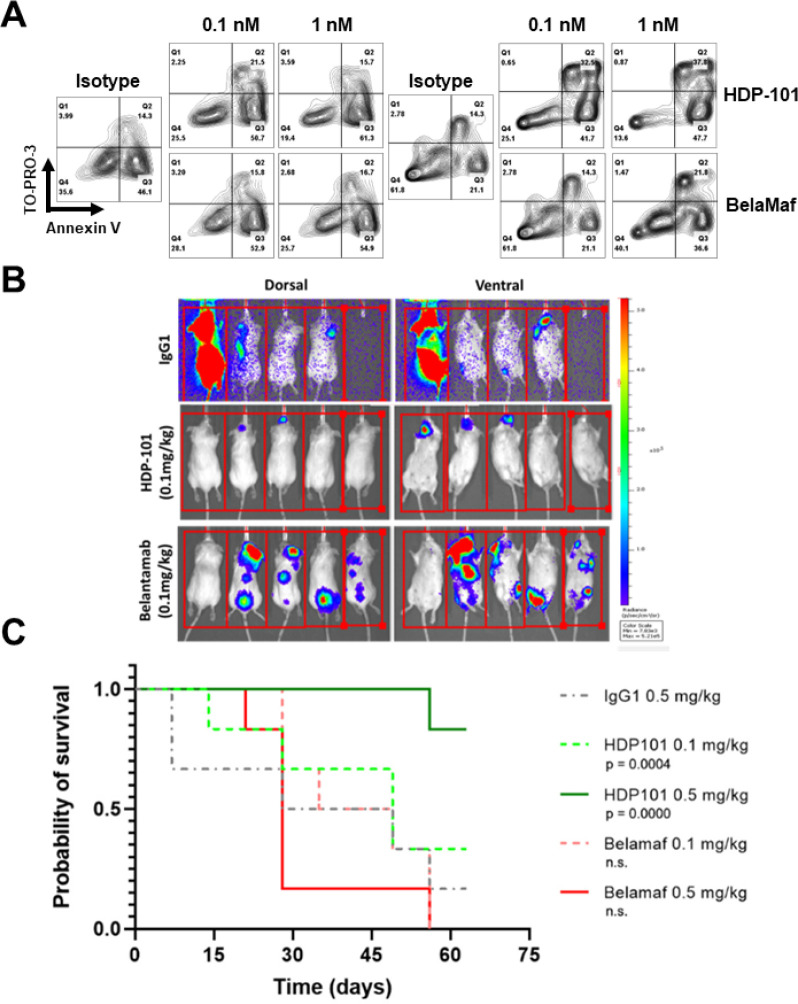
Comparative efficacy of HDP-101 and belantamab mafodotin. Primary samples from patients with relapsed/refractory myeloma were purified to isolate CD138^+^ cells, and then exposed to an isotype control antibody at 1 nM, or either belantamab mafodotin or HDP-101 at the 0.1 or 1.0 nM. They were then analyzed after dual TO-PRO-3 and Annexin V staining (**A**) by flow cytometry to detect apoptotic cells. NOD/SCID mice bearing systemic xenografts of MM1.S *TP53* KO/*POLR2A* KD cells received a single dose of an isotype control, or either HDP-101 or belantamab mafodotin at 0.1 or 0.5 mg/kg, as indicated. Disease was then monitored by whole animal *in vivo* imaging (**B**), and survival curves are shown for all groups (**C**) over 60 days of monitoring. Survival probability showed a significant improvement for HDP-101 at both the doses of 0.1 mg/kg (p=0.0004) and 0.5 mg/kg (p=0.00001), but not for belantamab.

Belantamab mafodotin (BelaMaf), a BCMA-targeted ADC linked to monomethyl-auristatin F, was the first drug in its class to achieve a regulatory approval for myeloma. However, more recently, it was withdrawn as a randomized study of this agent did not show sufficient superiority compared to a standard of care. Given the encouraging activity of HDP-101, we compared it directly to BelaMaf in both our *in vitro* and *in vivo* model systems. In the former, HDP-101, an ADC with a drug:antibody ratio (DAR) of 2 (24), more potently reduced viability of all of the myeloma cell lines we tested at 48 hours than was the case for BelaMaf (Table S2), which has a DAR of 4. At 96 hours, HDP-101 showed both greater overall efficacy than BelaMaf in our H929- and MM1.S-based WT, *TP53* KO, *POLR2A* KD, and dual KO/KD models, as well as preferential activity in the del17p dual knockout cells (Table 2). Next, we tested primary patient-derived samples, and found that HDP-101 induced a greater loss of viability than did BelaMaf at two different concentrations (Fig. 7A). Finally, we prepared a xenograft based on the del17p MM1.S dual *TP53* KO/*POLR2A* KD model, and treated these with a single dose of isotype control, or either 0.1 or 0.5 mg/kg of BelaMaf or HDP-101. This dose was selected to produce sub-total myeloma cell killing by HDP-101 to allow for better comparisons between the activity of the two ADCs. At day 60 (Fig. 7B), control mice had substantial disease burden that was reduced only modestly by BelaMaf at either of the two doses (Fig. 7C). In contrast, a statistically significant tumor growth inhibition was seen at both doses of HDP-101 (Fig. 7C) which, as expected, was greater at the higher dose, where some mice had no measurable tumor (Fig. 7B).

## Discussion

Outcomes for myeloma have significantly improved through the development of several now widely used drug classes and, as a result, the median overall survival of most patients treated with modern induction regimens is up to 10–15 years or more from diagnosis (39, 40, 41). However, there remains a high-risk group who still have substantially shorter remission durations despite the use of these therapies (7). Patients whose disease harbors del17p have consistently been identified as one of these groups, and one study noted that this may especially be the case in those with biallelic *TP53* inactivation, or so-called “double hit” myeloma (16). More recently, a second study confirmed this finding in patients treated with intensive therapy among whom “double hit” patients had a median survival of 36 months versus 152.2 months for controls (42), and even a single del17p conferred an inferior prognosis, with a median survival of 52.8 months. It is likely that these numbers will be improved by more novel therapies that induce deeper levels of remission, including those that target BCMA, but advances that leverage an understanding of the unique biology of each high-risk molecular subtype could be especially of benefit.

In light of the above, we studied HDP-101, a BCMA ADC conjugated to an optimized α-amanitin, with the rationale that del17p could produce haploinsufficiency of *POLR2A*, which is located nearby *TP53*, and heighten sensitivity to α-amanitin. Consistent with this possibility, del17p was associated in primary samples with reduced mRNA and protein levels of POLR2A, and HDP-101 showed enhanced efficacy against del17p models with biallelic *TP53* loss and *POLR2A* knockdown (Fig. 2). Mechanistically, HDP-101 reduced mitochondrial transmembrane potentials and induced apoptosis in association with activation of the ER and mitochondrial UPRs and induction of ICD (Figs. 2–4). Moreover, HDP-101 worked in concert with gamma-secretase inhibitors (Fig. S7) and overcame adhesion-mediated drug resistance (Figs. 1C, S2), confirming earlier studies (25, 26). Importantly, HDP-101 retained activity in models of acquired conventional and novel drug resistance (Table S3), indicating promise for activity in the relapsed/refractory setting where there is an unmet medical need. A single, well-tolerated dose of HDP-101 rapidly reduced disease burden to undetectable levels *in vivo* (Fig. 5), and relapse was not noted over a 100-day follow-up period even in the del17p models, which showed more aggressive growth without treatment. Importantly, HDP-101 showed enhanced efficacy compared with BelaMaf (Table 2, Fig. 7), the only other BCMA-targeted ADC to have achieved a regulatory approval endpoint. Notably, BelaMaf was recently withdrawn from the market after a randomized study showed a lack of sufficient superiority to a control treatment in the setting of relapsed/refractory myeloma, supporting the possibility that more potent agents like HDP-101 are needed.

ADCs with microtubule-targeting agents such as BelaMaf’s auristatins work well against proliferating cells (2) while amanitins can target quiescent cells (25), and may thus have an advantage against hypo-proliferative tumors like myeloma, as well as against myeloma-initiating or stem-like cells. The latter was supported by our studies of ALDH-positive clonogenic cells (Fig. S9), which were equally sensitive to HDP-101 compared with ALDH-negative cells. This ability to impact both committed plasma cells and their precursors may in part underlie the ability of HDP-101 to eradicate myeloma in *in vivo* models, including those with del17p, and to show greater efficacy than BelaMaf pre-clinically. It is interesting in this regard to note that one recent study described *TP53* loss as contributing to the tumor-initiating and drug resistance potential of clonogenic myeloma cells through activation of the Notch signaling pathway and upregulation of inhibitor of DNA binding (ID1/ID2) genes (43).

Another interesting aspect of HDP-101’s mechanism of action was its strong ability to induce immunogenic cell death, which was notable *in vitro* (Fig. 4). Moreover, rechallenge of previously treated mice at day 100 with new aliquots of tumor cells was not successful in producing tumor re-engraftment (Fig. 6). While this could theoretically be due to the presence of persistent levels of HDP-101 that retain anti-tumor activity, this seems unlikely since the clearance of ADCs is remarkably rapid in NSG mice based on prior studies showing a half-life of 1.4 days for an anti-CD30 construct (44). Furthermore, engraftment was enhanced by depletion of NK cells and macrophages using an anti-Asialo-GM1 antibody while this was not the case for control serum, undermining the possibility that this was due to remaining drug. These findings, which suggest the involvement of an anti-tumor effect from residual innate immune system cells in these mice, deserve follow-up in human models, since this agent is currently undergoing Phase I testing (45), to determine the immunologic effects of this drug in patients.

## Conclusions

The novel BCMA-targeted ADC HDP-101 showed robust anti-myeloma efficacy against both *in vitro* and *in vivo* models of multiple myeloma, overcame conventional and novel drug resistance, and induced both direct pro-apoptotic and immunogenic cell death mechanisms. Importantly, through its amanitin-based warhead, HDP-101 showed a uniquely enhanced efficacy against models of del17p myeloma which harbor haplo-insufficiency of its target, POLR2A. Since del17p represents a high-risk myeloma subtype that typically has an inferior benefit from other therapeutics, HDP-101 could represent an important advance in this unmet medical need population, and Phase I testing is currently underway.

## Figures and Tables

**Table 1:** Hallmark gene sets identified from the Molecular Signatures Database using gene expression data from myeloma cells exposed to HDP-101

**Gene Sets Up-regulated**
HALLMARK_G2M_CHECKPOINT
HALLMARK_E2F_TARGETS
HALLMARK_IL2_STAT5_SIGNALING
HALLMARK_UNFOLDED_PROTEIN_RESPONSE
HALLMARK_HYPOXIA
HALLMARK_MTORC1_SIGNALING
HALLMARK_MYC_TARGETS_V2
HALLMARK_ANDROGEN_RESPONSE
HALLMARK_MYC_TARG ETS_V1
HALLMARK_GLYCOLYSIS

**Table 2:** Median inhibitory concentrations (pM) for H929 and MM1.S-based myeloma cell line models treated with belantamab mafodotin or HDP-101 for 96 hours*

	HDP-101		BelaMaf	
	H929	MM1.S	H929	MM1.S
**TP53 WT/POLR2A WT**	155 ± 0.008	243 ± 0.04	1,157 ± 0.2	2,625 ± 0.4
**TP53 KO/POLR2A WT**	114 ± 0.01	270 ± 0.05	1,296 ± 0.1	3,145 ± 0.6
**TP53 WT/POLR2A KD**	29 ± 0.01	109 ± 0.01	1,269 ± 0.2	2,686 ± 0.5
**TP53 KO/POLR2A KD**	13 ± 0.005	75 ± 0.01	922 ± 0.08	4,096 ± 1.1

Abbreviations: BelaMaf, belantamab mafodotin; KD, knock down; KO knock out; POLR2A, RNA polymerase II subunit alpha; TP53, p53 tumor suppressor; WT, wild type
